# A Real-Time Optical Tracking and Measurement Processing System for Flying Targets

**DOI:** 10.1155/2014/976590

**Published:** 2014-04-02

**Authors:** Pengyu Guo, Shaowen Ding, Hongliang Zhang, Xiaohu Zhang

**Affiliations:** ^1^College of Aerospace Science and Engineering, National University of Defense Technology, Changsha 410073, China; ^2^Hunan Provincial Key Laboratory of Image Measurement and Vision Navigation, National University of Defense Technology, Changsha 410073, China

## Abstract

Optical tracking and measurement for flying targets is unlike the close range photography under a controllable observation environment, which brings extreme conditions like diverse target changes as a result of high maneuver ability and long cruising range. This paper first designed and realized a distributed image interpretation and measurement processing system to achieve resource centralized management, multisite simultaneous interpretation and adaptive estimation algorithm selection; then proposed a real-time interpretation method which contains automatic foreground detection, online target tracking, multiple features location, and human guidance. An experiment is carried out at performance and efficiency evaluation of the method by semisynthetic video. The system can be used in the field of aerospace tests like target analysis including dynamic parameter, transient states, and optical physics characteristics, with security control.

## 1. Introduction

The performance testing of flying targets like aircraft and missile is an important process in their civil or military production. The main testing item is trajectory measurement, including dynamic parameters (such as pose, velocity, and acceleration) and optical physics characteristics analysis (such as infrared radiation, flame spectrum, and luminance brightness). Optical tracking and measurement method is widely adopted in testing process, because optical sensor is passive, of low power consumption, and of noncontact; in addition, optical images are objective and informative. Because of targets' high maneuver ability and large cruising range, the observation environment is extreme, including target changes (such as pose, shape, scale, and motion blur) and circumstance changes (such as illumination and occlusion), but we still need high precision in performance analysis and high efficiency for security control. So an automatic and accurate interpretation method to conquer these extreme conditions is critical in real-time processing and this paper puts emphasis on the key components including foreground detection, online tracking, and feature location.

There are many worldwide famous video motion analysis systems, like TrackEye [1] from Sweden Image Systems cooperation, Visual Fusion [2] from America MIT, Movias Pro [3] from America NAC, and so forth. They all can provide transient state record, real-time image interpretation, and dynamic parameter estimation for flying targets using high-speed camera. But most of them depend on feature points or markers, whereas we care about the image interpretation for markerless targets with finite prior knowledge on the case that large sensor standoff distance and outdoor environment make a target region with a weak texture in the image.

This paper starts from the demand analysis of the optical tracking and measurement for flying targets.  Section 2 designs the architecture of distributed real-time processing system and introduces the compositions in brief. In Section 3 a detailed image interpretation method and a short parameter estimation method are presented.  Section 4 realizes the image interpretation subsystem and validates the performance by the experiment.  Section 5 draws the conclusion.

## 2. Architecture

**Figure 1:**
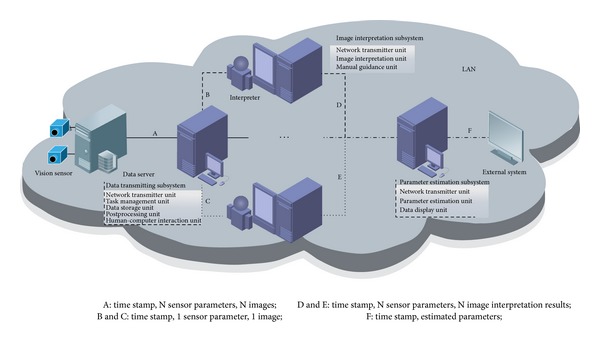
Architecture of distributed optical tracking and measurement processing system.

The real-time processing of optical tracking and measurement system has the characteristics of high efficiency, strong parallelism, and rigorous time series. As Figure 1 shows, the system adopts distributed architecture in terms of load balancing to improve efficiency and scalability, which is loosely decomposed into data receiving, image interpretation and parameter estimation three subsystems according to the course of first interpretation and then estimation. In view of the variety of different observation platforms and high computation resource demand of multisite synchronous interpretation, the parallel processing is needed which makes each interpretation subsystem accomplish one site task and the number of observation platforms decides the number of interpretation subsystems. The system communicates by network whose protocol is TCP/IP. Data server provides the data of the system via link A, which distinguishes data from different moments and sensors by time stamp and sensor serial number, and the results of estimation are exported to the external system for analysis and display.

It can provide two kinds of work modes including real-time and post-processing. Data transmitting subsystem is the manager of the whole system, which receives interpretation task data package from data server by link A, splits the package according to the sensor serial number to send each package to the subsystem by link B or C, and records the flying scene grouped by task in the database for postrecurrence and interpretation; image interpretation subsystem picks up the data package from data transmitting subsystem, extracts the image in the package to realize one site interpretation which can be guided by interpreter when the result is abnormal, and integrates the interpretation result with other information in the package to send it to the estimation subsystem through link D or E. Parameter estimation subsystem decides whether the data is from one site or multisite by time stamp alignment to select one site or multisite measurement manner automatically and send results to the external system for further analysis.

## 3. Real-Time Processing

The difficulty of real-time processing is accuracy and efficiency of the image interpretation, because parameter estimation methods during real-time or post-processing are nearly the same. So in this chapter, we propose a detailed real-time image interpretation method and make a brief introduction about parameter estimation.

### 3.1. Image Interpretation

Image interpretation is an image understanding process, which needs to detect the foreground target, track it online, and locate the feature for parameter estimation during real-time processing. A human guidance policy is essential on account of more false positives and false negatives during long-term flying.

#### 3.1.1. Foreground Detection

Automated systems need to use some form of foreground detection mechanisms to identify the target region to be tracked. Foreground detection can be divided into two types including single frame detection and sequence frames detection according to the number of used frames. Single fame detection is a visual object recognition problem [5] which needs the target prior appearance information from offline training and online update; sequence frames detection is a change detection problem which uses background subtraction like parameter estimation method GMM [6] or nonparameter estimation method VIBE [7] for fixed field of view and adopts a interframe registration such as optical flow [8] to describe motion information followed by a trajectory analysis based on tensor voting [9] or epipolar geometry [10] for varied field of view. Here we propose a simple detection algorithm which combines two-frame motion segmentation with one-single shape recognition.

**Figure 2:**
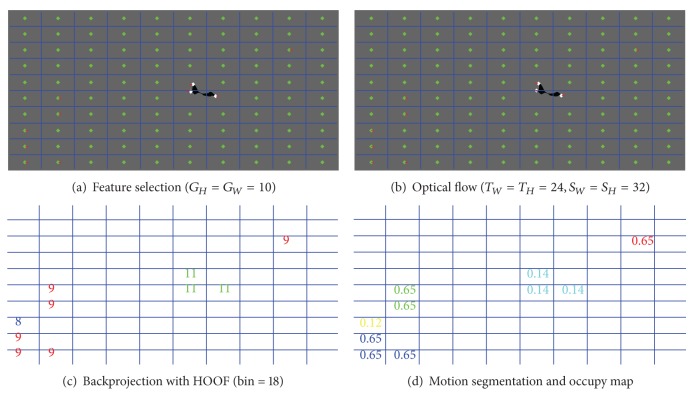
Motion detector.

The motion detector relies on two-frame optical flow filed. Many methods use a pixel-level analysis which is of high computational cost. In view of the scene whose background is nearly a plain region like sky or gobi in flying target tests, we downsample the image with *G*_*W*_ × *G*_*H*_ grids which is marked with one pixel in each grid. The pixel *P* may be a strong KLT feature point or a central point. If it is a KLT point, a pyramid Lucas-Kanade is used to track it; otherwise a pyramid NCC can be used to match the grid with a template size *T*_*W*_ × *T*_*H*_ and a search range *S*_*W*_ × *S*_*H*_. The feature selection is shown in Figure 2(a) with optical flow estimation *f*(*u*, *v*, *t*) in Figure 2(b) where white points are KLT feature points, green ones are central points, and red lines are optical flow. The orientation *θ* ∈ [0,2Π) and magnitude *m* of *f* can be computed in the following:

(1) $$\begin{array}{c} \theta =\text{ta}{\text{n}}^{-1}\left(\frac{v}{u}\right),\;\;m=\sqrt{u^{2}+v^{2}}, \end{array}$$

which can be used to count the histogram of optical flow orientation noted as HOOF assigning a weight *m* according to the following:

(2) $$\begin{array}{c} h_{j}=\sum_{i\in P\;}\sum_{j=1}^{\text{bin}}\left\lfloor \cos\left\lfloor \mathrm{sgn}\left(\frac{\theta_{i}}{\left(2^{*}\pi /\text{bin}\right)}-j\right)\right\rfloor \right\rfloor *m_{i}, \end{array}$$

where *P* is the set of sampling pixels, sgn is the sign function, and bin is the capacity of *h*. The normalized *h* is in (3). In light of *h*, a backprojection is shown in Figure 2(c) without stationary sampling points.  Figure 2(d) shows the motion segmentation result with occupy map based on HOOF where the same color means an identical motion region. Because the target may be a small part of the image which provides little motion information to the total optical flow field, we concern the connected region more than occupy value and tend to select larger occupy value when there are multiple connected regions

(3) $$\begin{array}{c} h_{j}=\frac{h_{j}}{\sum_{j=1}^{\text{bin}}h_{j}}. \end{array}$$

The appearance detector depends on the shape information, because the image intensity is varying along the observation distance, and the shape is more robust feature which is affected by visual angle and acquired easily. In addition, the observation distance is larger than the target dimension, so a projective transformation can be approximated by an affine warp.

**Figure 3:**
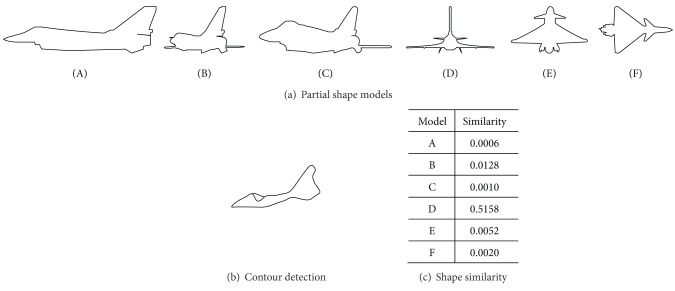
Appearance detector.

As in Figure 3(a) we render partial 2D aircraft shape models at some step according to the 3D model from Trimble 3D warehouse [11] and the visual angle. The affine-invariant boundary moment [12] in (4) is adopted to represent the shape which can bear small quantity of variable visual angle and noise,

(4) I1=(n20n02−n112)n004,I2=(n302n032−6n30n21n12n03+4n30n123   +4n213n03−3n212n122)×(n0010)−1I3=(n20(n21n03−n122)−n11(n30n03−n21n12)   +n02(n30n12−n212))×(n007)−1,

where *n* is the normalized boundary central moment. Compute the boundary moment *I* ^*B*^(*m*) of the *m*th target shape model *C* ^*B*^(*m*) offline noted as the set (*t*, *v*, *k*, *l*, *p*, *q*, *I* ^*B*^), where (*t*, *v*) is the sample identifier, *t* is the target type, *v* is the visual angle, the number of contour sampling points *p* is *k*, the number of predefined interest points *q* is *l*, and *I* ^*B*^ is the boundary moment descriptor. Calculate the boundary moment descriptor *I* ^*R*^(*n*) of the *n*th target contour *C* ^*R*^(*n*) online like Figure 3(b), and the similarity metric between *C* ^*B*^(*m*) and *C* ^*R*^(*n*) by a revised chi-square distance *d*(*m*, *n*) in (5) for the value of *I* ^*B*^ or *I* ^*R*^ may be nonpositive,

(5) $$\begin{array}{c} d\left(m,n\right)=\left|\frac{1}{2}\sum_{i=1}^{3}\frac{\left[I_{i}^{B}\left(m\right)-I_{i}^{R}\left(n\right)\right]}{I_{i}^{B}\left(m\right)+I_{i}^{R}\left(n\right)+\varepsilon }\right|, \end{array}$$

where *ε* is a small amount and less *d* means more similar. The detection is a nonminimum suppression process and a similarity metric between *C* ^*R*^(*n*) and *C* ^*B*^(*m*) is shown in Figure 3(c). The association of motion detector and appearance detector is easy, which outputs the biggest intersection as the target region with a bounding box definition.

#### 3.1.2. Target Tracking

Tracking must have the function of detection and on-line learning, because flying targets may leave/reenter the field-of-view and gradually change the pose. TLD [13] is a novel framework based on semisupervised learning which decomposes the tracking task into tracking, detection, integration, and learning four components. We improve it from adaption and efficiency for real-time application and propose an AA-TLD [14] as an acronym for adaptive and accelerated tracking-learning-detection.

**Figure 4:**
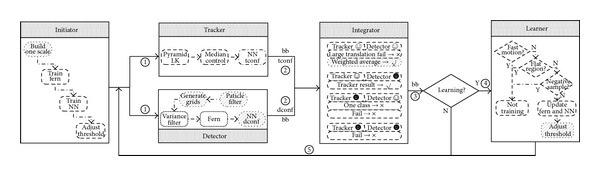
Flowchart of AA-TLD for target tracking. The marks white smile face and black smile face separately stand for success and failure symbols. The notations *√* and × are learning flags, which mean learning or not.

Figure 4 depicts the workflow of AA-TLD whose solid rectangle is a component, dashed rounded rectangle is a unit, and shaded dashed rounded rectangle is the modified unit. According to the bounding box of the target from foreground detection, initiator builds only the current scale space of the target with a sampling step and trains an initial fern detector and a NN detector according to a handful of samples with a threshold adjustment by cross-validation.

Unlike TLD's sequential execution, AA-TLD parallelizes tracker and detector to enhance the efficiency for independence by OPENMP. The tracker is the same with TLD, which uses median flow based on discrete sampling points in the target region and can be substituted with other easy tracking methods. The detector is realized in the particle filter framework to generate new scale scanning grids online according to the predicted scale and uses the same cascade process but fixes the number of the positive and negative samples which ensures a constant retrieving time by ordering the sample contribution ratio *c* as (6), where *s* is the positive or negative sample, *b* is the potential target grid, and *S* is the similarity metric in TLD

(6) $$\begin{array}{c} c\left(s_{i}\right)=\sum_{t=1\;}^{T}\sum_{g=1}^{G}S\left(b_{tg},s_{i}\right)\;s_{i}\in \left\{p,n\right\}. \end{array}$$

TLD associates the results of tracker and detector by weight-average which assigns the weight of tracking result 10, and each detection result 1. Maybe it is not convincing because the results are from different scales but are assessed by normalized correlation coefficient (NCC). AA-TLD adopts a weight computing method considering both NCC and scale size according to [15] as (7), where *r* is NCC, *n* is the number of pixels, and *ε* is a small quantity

(7) $$\begin{array}{c} \frac{0.5\ln\left(1+r\right)/\left(1-r+\varepsilon \right)}{\sqrt{1/\left(n-3\right)}}. \end{array}$$

**Figure 5:**
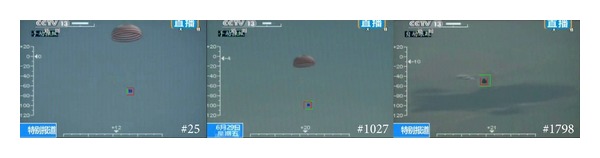
Tracking snapshots of Shenzhou IX. True value, the result of TLD, and the result of AA-TLD are, respectively, marked with blue box, green box, and red box.

**Figure 6:**
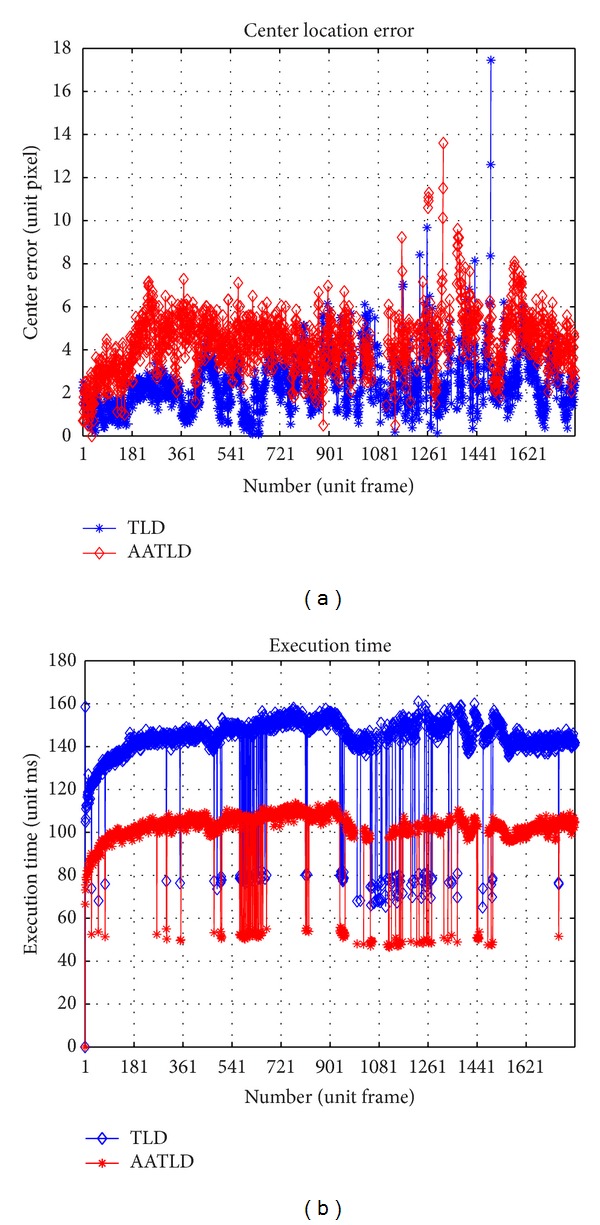
Performance contrast plots on center location and execution time of Shenzhou IX.

The learning stage of TLD only puts emphasis on updating the feature library by PN learning but not adjusting the threshold which is also important in decision process. AA-TLD adds a distance metric parameter adjustment component to online update the threshold by cross-validation like initiator. Partial tracking results of Shenzhou IX datasets which are shown in Figures 5 and 6 show the performance contrast details. We can conclude that AA-TLD is faster than TLD but less accurate than TLD at center location which is all right for the emphasis is an initial target region for feature location.

#### 3.1.3. Feature Location

The feature can be contour, line, or point which provides image coordinates to parameter estimation. Target tracking provides a bounding box of the target, and locating the feature in the bounding box will bring more accurate results than the total image.

The contour can be detected by a real-time approximate level-set method in [16]. The axis and the edge line are the major line feature in the image. After a state-of-the-art linear-time line segment detector LSD [17], clustering and additional criterion can be used to locate these edge line features. The axis can be extracted using moment of inertia of the target region or the halving line of two edge line segments.

The feature point can be centroid or interest points. The priority of the centroid computing is locating target pixel sets which can be acquired by contour scanning or saliency detection [18]. There are two kinds of interest points. One may be corners or blob-like points and can be established stereo correspondence by image matching like in [19]; the other can be the projection of the physical points as in [20, 21] with a prior knowledge of the target like 3D or 2D model. Here we propose simple and fast multiple interest physical points locating method based on ICP [22] with 2D shape models in Algorithm 1, which can offer image coordinates to pose estimation based on points.

**Figure 7:**
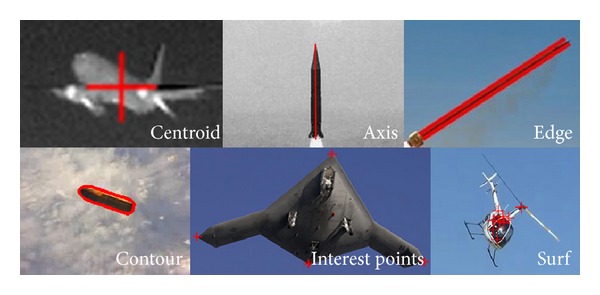
Examples of feature location.

Some examples of feature location are shown in Figure 7. Different features adapt to different targets and observation conditions.

#### 3.1.4. Human Guidance

**Figure 8:**
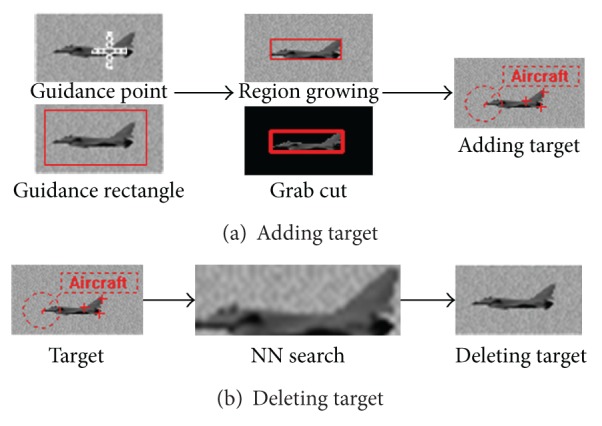
Guidance process.

For correctness and efficiency of the system, when interpretation error occurs, the interpreter instantly provides guidance information which can be a simple seed point or a coarse rectangle region by human-computer interaction. The guidance does not interrupt the continuous running; when the system receives the guidance information, it will seek the bounding box of the target using region growing algorithm based on a guidance point or search it using grab cut [23] based on a guidance rectangle and learn the region feature to update the sample library at the next frame. The update strategy contains adding the feature which puts the positive sample into the library and deleting the feature that removes the similar sample with the guidance region from the library by nearest neighbor (NN) search as shown in Figure 8.

### 3.2. Parameter Estimation

Optical sensor can provide more appearance information like color, texture, and gradient than radar which relies purely on position and motion information [24]. Optical measurement can supply not only dynamic parameter estimation but also optical characteristic analysis.

#### 3.2.1. Dynamic Parameter Estimation

Different observation manners and image features need different parameter estimation methods. The conventional observation apparatus for flying target tests is optoelectronic theodolite for long-distance measurement or high-speed camera for close-range measurement.

The measurement based on optoelectronic theodolite [25] can realize one-site localization, because the apparatus outputs the angular altitude, azimuth angle, and slope distance of the target by missing distance interpretation of one point standing for the target. And multisite triangulation will bring higher precision than one-site localization. If there is no slope distance information like high-speed camera observation, one site interpretation with material point hypothesis cannot locate the target, and the triangulation measurement [26] is necessary.

One point interpretation cannot estimate the attitude. But multiple points from single sensor with known correspondence between object points and image points is a classical PNP problem which estimates the pose like [27, 28]. The axis is the common line segment feature for rigid rotation, which can be used to locate with triangulation and estimate the pose in [29, 30]. With 3D model and the contour in the image of the target, model-based pose tracking method can be used to estimate the pose, like PWP3D [20]. The trajectory, velocity, and acceleration can be computed with time information.

#### 3.2.2. Optical Characteristic Analysis

**Figure 9:**
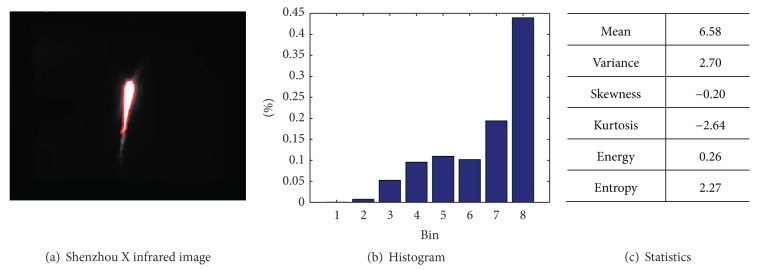
Example of optical characteristic analysis.

The analysis of optical physics characteristics about infrared radiation, flame spectrum, and luminance brightness needs the silhouette *S* of the target in the image. The histogram of the intensity of the pixels inside *S* represents the characteristics, and the histogram *h* is defined as follows:

(8) $$\begin{array}{c} h=\left\{h_{k}\;|\;h_{k}=\frac{N_{k}}{N}\right\}\;k=1,2,3,\ldots ,L, \end{array}$$

where *N* is the number of pixels inside *S*, *L* is the intensity bins, and *N*_*k*_ is the number of pixels which belongs to the *k*th intensity bin. The statistical optical characteristics can derive from the mean, variance, coefficient of skewness, coefficient of kurtosis, energy, entropy, and other criteria of *h*.  Figure 9 illustrates an example of optical characteristic analysis.  Figure 9(a) shows an infrared image of Shenzhou X emission marked with the contour, whose histogram is in Figure 9(b) with intensity range from 0 to 255 and 8 intensity bins.  Figure 9(c) presents the statistical analysis.

## 4. Experiment

**Figure 10:**
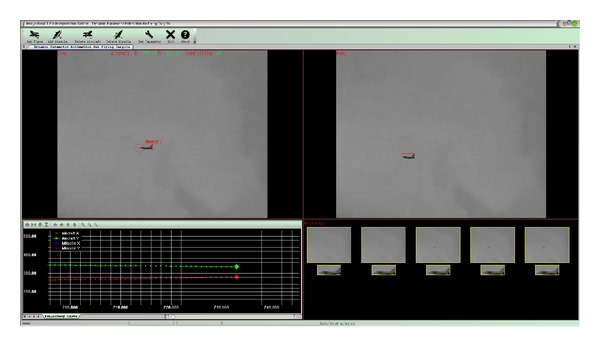
Image interpretation subsystem UI.

We test our real-time processing system at precision and efficiency based on the image interpretation subsystem, which is developed on VS2008 with VC++ and implemented on an Intel Core i5-3470 3.20 GHz CPU with 3.46 GB RAM/ Windows X86-32 bit machine.  Figure 10 shows the system UI including the menu bar and the windows. The menu bar consists of the human guidance buttons and the configuration button. The windows are the live image, the model image, the curve diagram, and the last five target regions.

### 4.1. Performance

**Figure 11:**
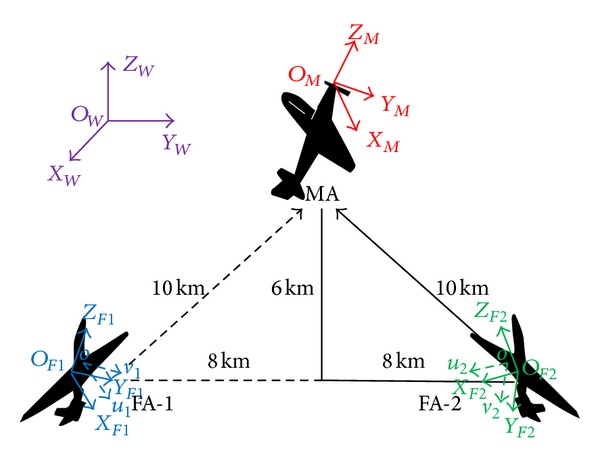
Supposed measurement scene.

Supposing the scene where two following aircrafts (noted as FA-1 and FA-2) are tracking and recording the measured aircraft (MA) during 80 s by a 25 Hz camera with focal length from 150 mm to 300 mm and pixel size 6 *μ*m shown in Figure 11, we generate 2000 digital gray scale image sequences with resolution of 720 × 576. Dynamic parameter estimation needs six reference coordinates, which are the world coordinate system *O*_*W*_-*X*_*W*_ *Y*_*W*_ *Z*_*W*_, the coordinate system of MA *O*_*M*_-*X*_*M*_ *Y*_*M*_ *Z*_*M*_, the camera coordinate system of FA-1 *O*_*F*1_-*X*_*F*1_ *Y*_*F*1_ *Z*_*F*1_ with the image coordinate system *o*-*u*_1_ *v*_1_, and the camera coordinate system of FA-2 *O*_*F*2_-*X*_*F*2_ *Y*_*F*2_ *Z*_*F*2_ with the image coordinate system *o*-*u*_2_ *v*_2_. We first locate the four interest points of reference points of MA in the image coordinate then estimate the position and the attitude of MA in *O*_*W*_-*X*_*W*_ *Y*_*W*_ *Z*_*W*_ with known camera extrinsic parameters and coordinates of reference points.

#### 4.1.1. Image Feature Location Precision

**Figure 12:**
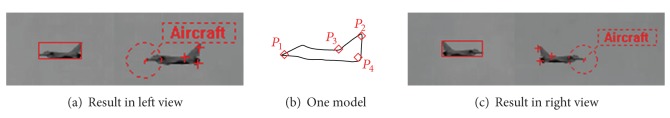
Detection and tracking results based on one model.

The four predefined interest points of the aircraft model are shown in Figure 12(b) marked with *P*_1_ to *P*_4_. Figures 12(a) and 12(b) are the foreground detection result signed with bounding box and online interpretation result marked by crosses from two views.

**Figure 13:**
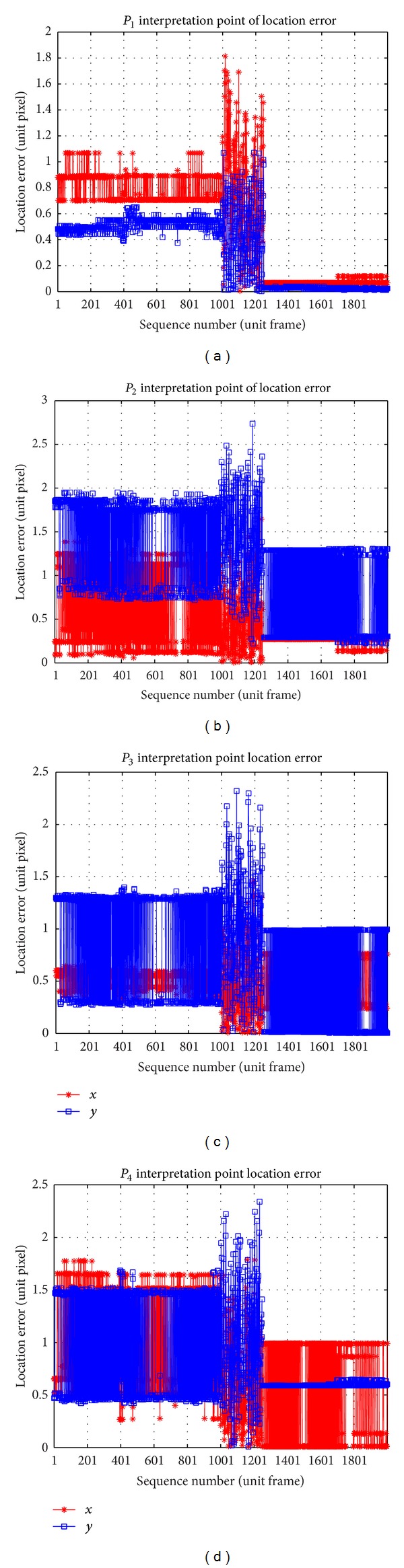
Plots of location precision of multiple interest points for left view.

The location precision of multi-interest points is shown in Figure 13. The true value coordinate of the target is (*x*_*ti*_^*b*^, *y*_*ti*_^*b*^), and the interpretation coordinate is (*x*_*ti*_^*r*^, *y*_*ti*_^*r*^), where *t* is the sequence number of frames, *i* is the label of the interpretation points, *M* is the amount of interpretation frames, and *N* is the number of interpretation points. Here *N* equals 4, and location standard deviation is (0.3, 0.5) pixel for left view and (0.6, 0.4) pixel for right view on the basis of the following:

(9) $$\begin{array}{c} \delta_{x}=\sqrt{\sum_{t=1\;}^{M}\sum_{i=1}^{N}\frac{\left(x_{ti}^{b}-x_{ti}^{r}\right)}{\left(MN-1\right)}}\;\;\delta_{y}=\sqrt{\sum_{t=1\;}^{M}\sum_{i=1}^{N}\frac{\left(y_{ti}^{b}-y_{ti}^{r}\right)}{\left(MN-1\right)}} \end{array}$$

#### 4.1.2. Dynamic Parameter Estimation Precision

**Figure 14:**
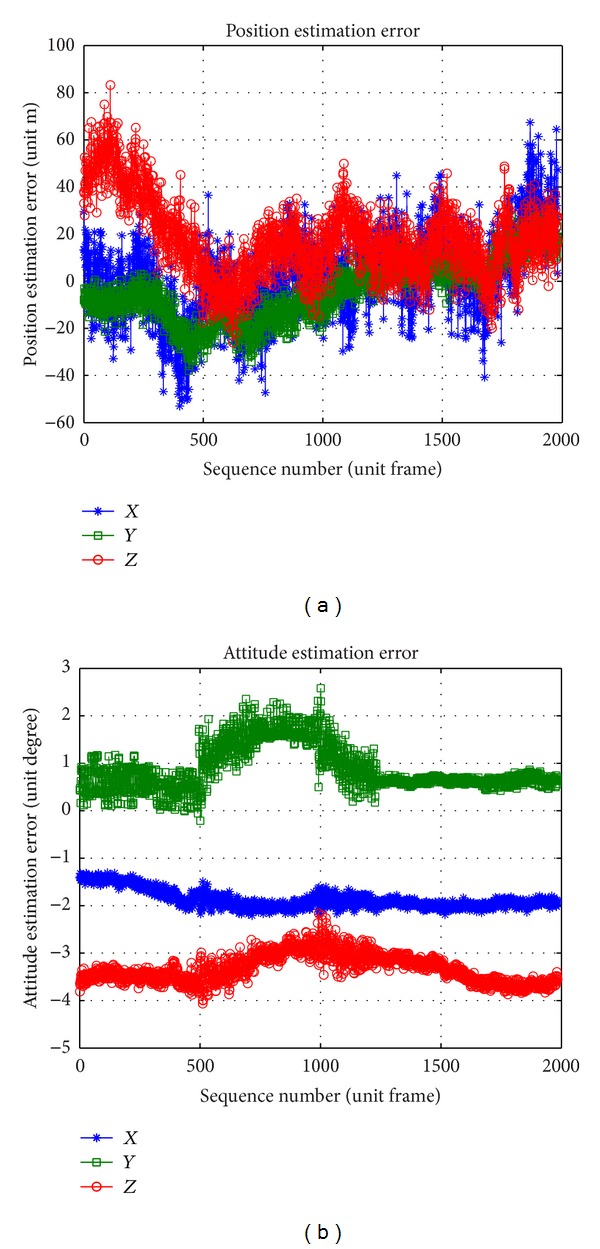
Plots of dynamic parameter estimation precision.

The measurement precision about position and attitude is shown in Figure 14. The position is estimated by two-view triangulation with standard deviation (18.0, 13.6, and 24.1) m, and the attitude is estimated by absolute orientation with standard deviation (1.88, 1.00, and 3.34)° in one experiment, when there are 0.1° system error with 0.05° standard deviation of rotation angle, 10 m system error with 2 m standard deviation of translation about camera extrinsic parameters, and 0.5 m object error. The excellent filter like Kalman can be used to estimate the velocity and the angular velocity with position, attitude, and time information.

### 4.2. Efficiency

**Figure 15:**
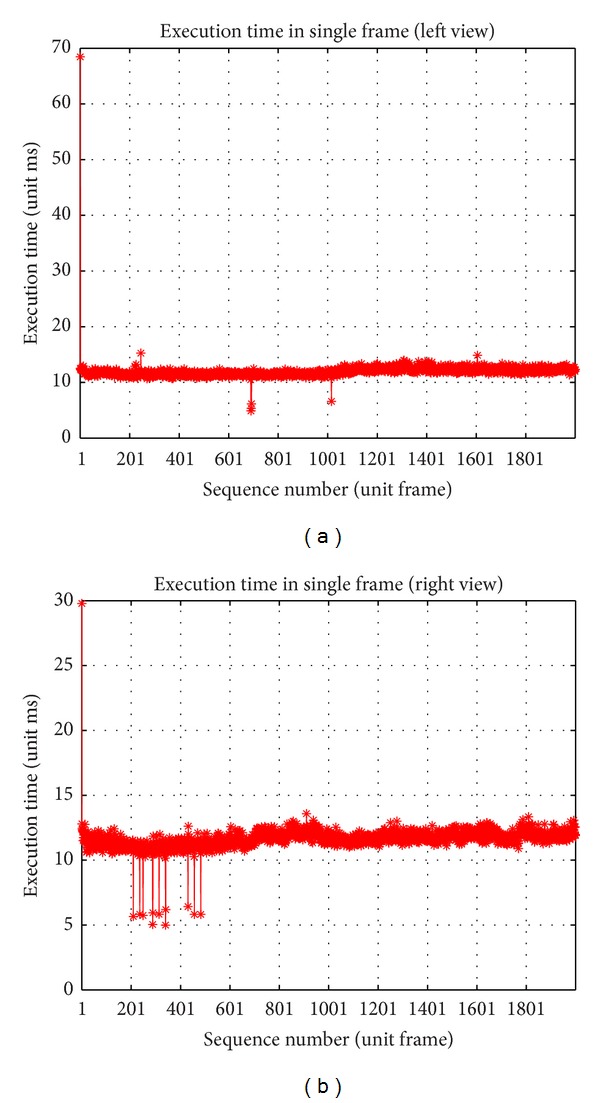
Plots of execution time.

Figure 15 shows the execution time of the single frame about 12 ms/frame, and more initialization time is required than online processing. The location of multi-interest points needs an iterative optimization; for real-time, we not only use the frequency limitation, but also build shape samples library with some fixed step to ensure a superior initial value. In our experiment, because of the pattern of side direction observation, the number of iteration is less than 2.

## 5. Conclusion

This paper proposes a real-time processing method of optical tracking and measurement system for flying targets with a detailed image interpretation method and a brief parameter estimation review and realizes a distributed real-time image interpretation and parameter estimation system. The simulated experiments validate the accuracy and the efficiency of the system. The Achilles' heel of the method and the system is not adapting to all the outdoor rigorous observation conditions, like uneven illumination and long-time occlusion, which seriously affect the feature location. Next we will put emphasis on robust feature location algorithms to improve the image interpretation precision and use more sophisticated real experiments for the system optimization. The system can be used to flying targets tests to meet the users' need of real-time interpretation.

## Figures and Tables

**Algorithm 1:**
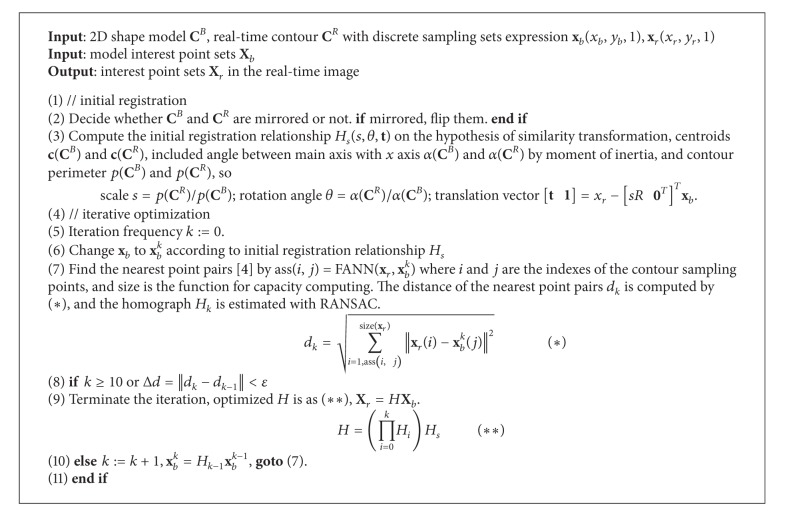
Multiple interest points location based on 2D contour model.
